# Endometrial mesenchymal stem/stromal cells: The Enigma to code messages for generation of functionally active regulatory T cells

**DOI:** 10.1186/s13287-021-02603-3

**Published:** 2021-10-09

**Authors:** Mehdi Aleahmad, Mahmood Bozorgmehr, Shohreh Nikoo, Alireza Ghanavatinejad, Mohammad-Reza Shokri, Samaneh Montazeri, Fazel Shokri, Amir-Hassan Zarnani

**Affiliations:** 1 Department of Immunology, School of Public Health, Tehran University of Medical Sciences, P.O. Box: 1417613151, Tehran, Iran; 2 Reproductive Biotechnology Research Center, Avicenna Research Institute, ACECR, Tehran, Iran; 3 Immunology Research Center, Institute of Immunology and Infectious Diseases, Iran University of Medical Sciences, Tehran, Iran

**Keywords:** MenSCs, Regulatory T cells, Proliferation, Endometrium, Pregnancy, Immunomodulation

## Abstract

**Background:**

Regulatory T cells (Tregs) play an important role in fine-tuning of immune responses and are pivotal for a successful pregnancy. Recently, the importance of mesenchymal stem cells in regulation of immune responses in general and Tregs in particular has been highlighted. Here, we hypothesized that menstrual stromal/stem cells (MenSCs) contribute to uterine immune system regulation through induction of functionally active Tregs.

**Methods:**

MenSCs were collected from 18 apparently healthy women and characterized. Bone marrow mesenchymal stem cells (BMSCs) served as a control. The effect of MenSCs on proliferation of anti-CD3/CD28-stimulated T CD4 + cells and generation of Tregs with or without pre-treatment with mitomycin C, IFN-γ and IL-1β was evaluated by flow cytometry. The potential role of IDO, PGE2, IL-6, IL-10, and TGF-β on proliferation of T CD4 + cells and generation of Tregs was assessed using blocking antibodies or agents. IDO activity was evaluated in MenSCs and BMSCs culture supernatants by a colorimetric assay. IL-10 and IFN-γ production in MenSCs-primed T CD4 + was measured using intracellular staining. To investigate the functional properties of Tregs induced by MenSCs, Treg cells were isolated and their functional property to inhibit proliferation of anti-CD3/CD28-stimulated PBMCs was assessed by flow cytometry.

**Results:**

According to the results, proliferation of T CD4 + lymphocytes was enhanced in the presence of MenSCs, while pre-treatment of MenSCs with pro-inflammatory cytokines reversed this effect. PGE2 and IDO were the major players in MenSCs-induced T cell proliferation. Non-treated MenSCs decreased the frequency of Tregs, whereas after pre-treatment with IFN-γ and IL-1β, they induced functional Tregs with ability to inhibit the proliferation of anti-CD3/CD28-stimulated PBMCs. This effect was mediated through IL-6, IL-10, TGF-β and IDO. IFN-γ/IL-1β-treated MenSCs induced IL-10 and IFN-γ production in CD4 + T cells.

**Conclusion:**

Collectively, these findings indicate that immunomodulatory impact of menstrual blood stem cells (MenSCs) on generation of Tregs and inhibition of T cells proliferation is largely dependent on pre-treatment with IFN-γ and IL-1β. This is the first report on immunomodulatory impact of MenSCs on Tregs and highlights the pivotal role of endometrial stem cells in regulation of local endometrial immune responses.

**Supplementary Information:**

The online version contains supplementary material available at 10.1186/s13287-021-02603-3.

## Background

Regulatory T cells (Tregs), as one of the main regulators of the immune system, play an important role in fine-tuning of immune responses and preventing autoimmunity. The presence of Tregs in the uterus and their positive impact in successful pregnancy have been fully understood [1, 2]. The frequency of circulatory Tregs in non-pregnant fertile women reaches to the highest level between days 9 to 13 of follicular phase and reduces during the luteal phase of the menstrual cycle [3], which is believed to be essential to prepare the uterus for encountering the paternal antigens and possible implantation [4]. Along with that, it is known that same alteration in Treg population can be observed in murine estrus cycle [5].

In very early stages of human pregnancy, systemic and local frequency of Tregs in decidua, lymph nodes and spleen is increased and reaches its peak on second semester of gestation [2, 6, 7]. Treg frequency is then diminished throughout the weeks prior to the childbirth and a clear decrease is seen after the delivery, even though it is still slightly higher than non-pregnant women [2, 8]. Frequency of murine endometrial Tregs peaks in the estrus phase and then declines during metestrus and remains low until the next estrus [5, 9]. In pregnant mice, Tregs are quickly recruited to uterus draining lymph nodes and are activated during the first 2 days after the implantation [1]. In the early stages of mouse pregnancy, the origin of accumulated Tregs in uterine draining lymph is thymus that will substitute for periphery in later pregnancy stages [10]. The importance of Tregs for successful pregnancy in mice is substantiated by exploiting an adoptive transfer model. In this model, transferring Treg-depleted T cell population to nu/nu BALB/c mice led to fetus rejection in allogeneic mating, whereas transferring total T cell population resulted in successful pregnancy [1].

Antigen-dependent and antigen-independent mechanisms contribute to induction of Tregs during pregnancy, resulting in immunological tolerance toward the fetus without endangering the integrity of the mother's immune system. The most important factors that trigger antigen-independent pathway of Tregs induction include estrogen [11], progesterone [12], PGE2 [13], TGF-β [14] and IDO. IDO is one of the known inhibitory factors secreted from different cells of the immune system and also MSCs, plays a vital role in pregnancy immune tolerance by metabolizing tryptophan at the maternal–fetal interface [15]. Sufficient amount of IDO in placenta and decidua is necessary to prevent rejection of fetal allograft and maintenance of normal pregnancy [16, 17] through induction of Foxp3^+^ regulatory T cells [18]. Furthermore, kynurenine as a tryptophan metabolite produced by IDO can suppress the immune system and inhibit T cell proliferation by induction of cell cycle arrest and apoptosis [19].

In human, a sharp increase in the number of Tregs in the follicular phase is strictly associated with high level of serum estradiol (E2) [3], which not only can affect Treg Foxp3 mRNA expression but also can cause elevation of chemokines responsible for recruitment of Treg cells to the uterus. In parallel, progesterone can induce the development of Tregs and suppress the differentiation of Th1 and Th17 cells [12, 20]. In the antigen-dependent pathway, the role of seminal fluid in keeping antigenic and environmental signals is quite essential [21]. Seminal fluid contains steroid hormones such as testosterone, estrogens and prostaglandin [22] along with TGF-b [23, 24], which induce uterine hypo-responsiveness to sperm alloantigen’s through induction of Tregs [21, 25].

Recently, the importance of mesenchymal stem cells in regulation of immune responses in general and Tregs in particular has been highlighted. Several studies have shown the ability of mesenchymal stem cells (MSCs) from different origins in induction of Tregs. BM-MSC is able to inhibit T cell proliferation and shift T cell profile toward Tregs [26, 27]. MSCs from other sources such as those derived from adipose tissue also mediate the generation of Tregs with immunosuppressive ability [28, 29]. As one of the most regenerative tissues in the human body, the endometrium provides a unique source of adult stromal/stem cells. The immunomodulatory capacity of endometrial stromal/stem cells (eMSCs) has not been fully elucidated. Reportedly, this cell type can inhibit the proliferation of mouse splenocytes stimulated with mitogen [30], and also, it has the capacity to recruit immature dendritic cells by different array of chemokines [31, 32]. eMSC population shed during menstruation into menstrual fluid. Menstrual blood stromal/stem cells (MenSCs) are newly identified adult stem cells with multi-lineage differentiation capacity [33–38]. In 2008, Murphy et al. proposed the immunomodulatory ability of MenSCs to suppress mixed lymphocyte reaction (MLR) and to attenuate pro-inflammatory responses [39], a finding that was supported by our investigations showing the suppressive effects of MenSCs on allogeneic MLR in a dose-depended manner [40]. These cells share phenotypic and functional properties with other MSCs, yet have their own specificities [41, 42]. Moreover, we also reported the potency of these cells to inhibit dendritic cell maturation [43]. In a recent report, we showed that MenSCs are able to modulate functional features of natural killer (NK) cells and induce a pregnancy-friendly phenotype in these cells [44]. Here, we hypothesized that MenSCs contribute to uterine immune system regulation through induction of functionally active regulatory T cells.

## Materials and methods

### Sample collection

Menstrual blood was collected from 18 apparently healthy women aging between 25 and 35 years after getting signed inform consent. We were not able to do all experiments on each MenSCs sample because experiments had to be performed on MenSCs with low passage number. Menstrual blood samples did not yield a sufficient number of cells suffice to do all experiments simultaneously. Indeed, doing all experiments at the same time was practically impossible. So, we did a part of the experiments on a set of samples and the rest of experiments with another sets. Nonetheless, not less than six samples were tested in any experiment. Participants had at least one live birth, no history of pregnancy complications including abortion and preeclampsia, no background diseases and no consumption of contraceptive, anti-inflammatory drugs, corticosteroids or vitamin D in the last three months before sampling. They also had negative test results for such blood transmittable viruses such as HCV, HBV, and HIV and had no sign or symptoms of endometriosis and autoimmune diseases. Menstrual blood sample collection was performed as we described recently [44] in the second day of menstruation using Diva Cup (Lunette, Stockholm, Sweden) and immediately transported to the laboratory in a cold chain. Bone marrow-derived mesenchymal stem cells (BMSCs) from four healthy donors were obtained from Avicenna Research Institute (ARI) biobank, and buffy coats were collected from Iranian Blood Transfusion Organization (IBTO).

### MenSCs isolation and culture

Isolation of MenSCs was carried out according to the protocol we described recently [45]. In brief, menstrual blood samples were washed and cultured in DMEM/F12 supplemented with penicillin–streptomycin and 10% fetal bovine serum (FBS) (Gibco, USA). After 48 h., non-adherent cells were removed and MenSCs were allowed to propagate. After confluency of about 80%, cells were detached using trypsin–EDTA (Gibco, USA) and stored at liquid nitrogen. BMSCs were cultured in the same condition as with MenSCs. All experiments were performed on MenSCs and BMSCs at passages below five.

### Multi-lineage differentiation and immunophenotyping of MenSCs

Differentiation of MenSCs toward osteocytes, chondrocyte, and adipocyte was carried out according to the protocols we published elsewhere [37, 38, 45]. Immunophenotype of MSCs was examined by flow cytometry using a panel of antibodies against mesenchymal and embryonic stem cells and hematopoietic cell markers (Table 1). All immunostainings were done by direct staining with fluorochrome-labelled antibodies except for Oct-4 which was done by two-step indirect intracellular staining using rabbit anti-human Oct-4 antibody (Abcam, UK) and FITC-conjugated goat anti-rabbit Ig (Abcam, UK). Signals were analyzed using Attune NxT flow cytometer (Thermo Fisher Scientific, USA) in reference to appropriate isotype controls.

**Table 1:** Antibody panel for immunophenotyping of MenSCs

Antibody	Fluorochrome	Clone	Company
Anti-CD9	FITC	M-L13	BD Biosciences
Anti-CD10	PE	H110a	BD Biosciences
Anti-CD29	PE	MAR4	BD Biosciences
Anti-CD34	FITC	581	BD Biosciences
Anti-CD38	FITC	HIT2	BD Biosciences
Anti-CD44	PE	515	BD Biosciences
Anti-CD45	PE	HI30	BD Biosciences
Anti-CD73	PE	AD2	BD Biosciences
Anti-CD105	PE	166707	BD Biosciences
Anti-CD133	PE	W6B3C1	BD Biosciences
Anti-Oct-4	–	Polyclonal	Abcam
Anti-SSEA-4	–	MC813-70	BD Biosciences

### T CD4 + isolation and MenSCs-T CD4 + co-culture

Peripheral blood mononuclear cells (PBMCs) were isolated from buffy coat using Ficoll-Hypaque density gradient medium (Biosera, USA). Upon harvesting and washing, CD4 + T cells were isolated from PBMCs using human T CD4 + cell Isolation kit (Miltenyi Biotec, Germany) according to manufacturer’s instruction. Isolated cells had viability and purity of > 95% as assessed by trypan blue dye exclusion and CD4 + staining by flow cytometry. For MenSCs-T CD4 + co-culture, 2 × 10^5^ freshly isolated CD4 + T cells were co-cultured with titrating amounts of MenSCs in 24-well plates (Corning, USA) in a total volume of 1 mL/well. Culture medium consisted of RPMI-1640 (Gibco, USA) supplemented with penicillin (100 μg/mL), streptomycin (100 IU/mL), L-glutamine (2 mM) (all from Sigma, USA), non-essential amino acids, sodium pyruvate, and 10% FBS (all from Gibco, USA). Several different settings were tested in MenSCs-T CD4 + co-culture. In one setting, MenSCs were remained untreated or mitotically inactivated with 25 µg/mL of Mitomycin C (Sigma, USA) for 1 h. before co-culturing. In parallel, to verify the effect of IFN-γ and/or IL-1β on MenSCs function, MenSCs were treated with either 25 ng/mL IFN-γ (BD Biosciences, USA) and/or 10 ng/mL IL-1β (BD Biosciences, USA) 48 h. prior to co-culture with T CD4 + cells. At initiation of the co-culture, T cells were activated with activation beads containing 5 µg/mL anti-CD3 and 5 µg/mL anti-CD28 at 4:1 ratio (CD4 + T cell to bead) according to manufacturer’s instruction (Miltenyi Biotec, Germany). In the third day of co-culture, half of the culture media was gently replaced with fresh RPMI-1640, containing 100 U/mL recombinant IL-2 (R&D systems, USA). In most settings, BMSCs were co-cultures in parallel.

### Measurement of IDO activity

A colorimetric method was used to determine the activity of IDO in MenSCs and BMSCs. MenSCs were treated with 100 ng/mL IFN-γ (BD Biosciences, USA) in the presence or absence of 10 ng/mL IL-1β (BD Biosciences, USA) or remained untreated. Similarly, BMSCs were treated with IFN-γ or remained untreated as a control. Untreated cells or those treated with 100 ug/mL L-Tryptophan (Trp) served as controls. The activity of IDO was measured as described earlier [46].

### Proliferation assay and Treg generation

To assess the effects of MenSCs on CD4 + T cells proliferation, CD4 + T cells were co-cultured in different settings as mentioned above. Before co-culturing, CD4 + T cells were washed and labeled with 5 μM 5,6-carboxyfluorescein diacetate succinimidyl ester (CFSE) (Molecular Probes, USA).

To investigate the effects of MenSCs secretome on CD4 + T cell proliferation, a transwell system (Corning, USA) was utilized. Briefly, IFN-γ/IL-1β-treated MenSCs were seeded in the lower chamber of a 24-well transwell plate. Later, CD4 + T cells were added into the upper chamber and plates were incubated for five days in incubator. In some settings, 1 mM 1-Methyl-DL-tryptophan (Sigma, USA) as IDO blocker, 20 mM Indomethacin (Sigma, USA) as PGE2 inhibitor, 10 µg/mL anti-IL-6 antibody (R&D systems, USA), 10 µg/mL anti-IL10 antibody (R&D systems, USA) or 10 µg/mL anti-TGF-β (R&D systems, USA) were added to the co-cultures in order to investigate the involvement of aforesaid molecules on modulatory effects of MenSCs on T cell proliferation or Treg generation. After 5 days, proliferation of CD4 + T cells and frequency of Tregs were assessed by flow cytometry.

### Isolation of Tregs and functional assays

To investigate the percentage and functional properties of Tregs induced by co-culture of CD4 + T cells with MenSCs, Treg cells were isolated from 5-day co-cultures by Treg isolation kit (Miltenyi Biotec, Germany) according to manufacturer’s instruction, and their functional property to inhibit stimulated PBMCs was assessed. For this purpose, PBMCs autologous to the Tregs were first stained with CFSE and added to the wells of 96-well U bottom plates (Corning, USA) (5 × 10^4^ cells/well/50 µL). Isolated Treg cells were then added to the wells at the ratios of 1:5 and 1:10 (Treg to PBMCs) in a volume of 50 µL/well. Finally, activation/expansion beads (Miltenyi Biotec, Germany) containing 5 µg/mL anti-CD3 and 5 µg/mL anti-CD28 at 1:2 ratio (bead to PBMC) were added to the wells, and total volume of each well was adjusted to 200 µL. In the second day of culture, 100 µL of culture medium was gently replaced with fresh culture medium containing 200 U/mL IL-2 (R&D systems, USA) and incubation was continued for 2 days at %CO2 incubator. Proliferation of PBMCs was then measured by flow cytometry.

### Flow cytometry

Flow cytometry experiments were performed to assess T cells co-cultured with MenSCs. The fluorochrome-conjugated antibodies included Alexa Fluor 647 anti-FoxP3 (BD Biosciences, USA), FITC anti-human IFN-γ, PerCP/Cy5.5 anti-CD4, APC anti-human IL-10, and PE anti-CD25 (All from Biolegend, USA) were employed. Antibodies were used at the concentrations recommended by the manufacturers. For assessment of cytokines, cells were treated with 50 µg/mL PMA (Sigma, USA), 1 µg/mL ionomycin (Sigma, USA) and 0.7 µg/mL Monensin (BD Biosciences, USA) 6 h. before staining procedure. For intracellular staining of Foxp3 and cytokines, transcription factor buffer set (BD Biosciences, USA) was used for cell permeabilization and fixation according to the manufacturer’s instruction. Cells were also stained with Live/Dead fixable near red fluorescent dye (Molecular Probes, USA) to separate the alive and dead populations before antibody-specific gating. The stained cell was then read by flow cytometer Attune NxT flow cytometer and analyzed by FlowJo software (FlowJo, LLC, USA).

### Statistics

All statistical analyses and group comparisons were performed using GraphPad Prism 8. Data were compared with the Mann–Whitney test. Descriptive data were reported as mean ± standard error of the mean (SEM). P values less than 0.0332 were interpreted as statistically significant. P values less than 0.0332, 0.0021, 0.0002, and 0.0001 were shown as *, **, ***, and ****, respectively.

## Results

### Workflow of the experimental design

**Fig. 1:**
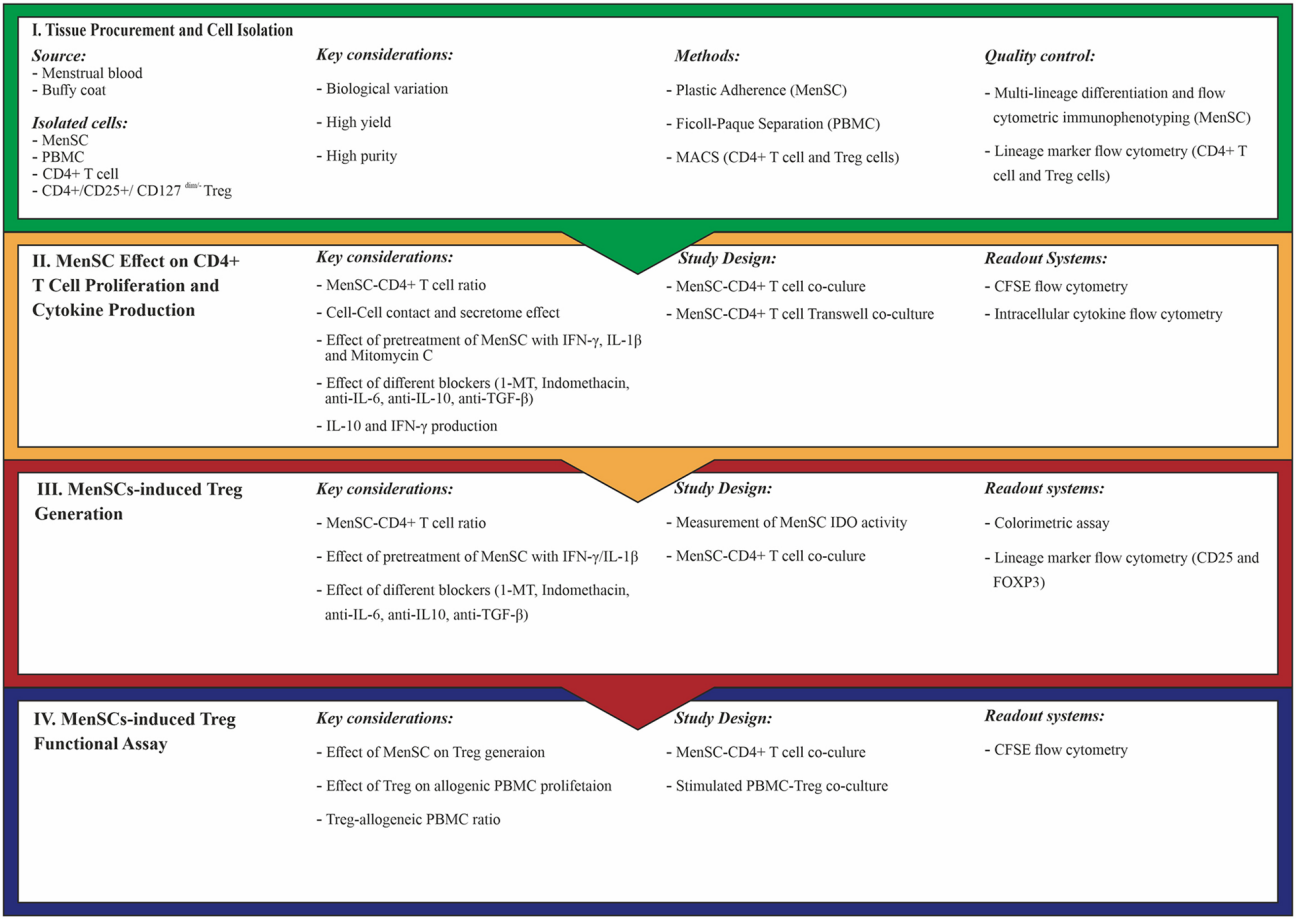
Workflow of the experimental design

Figure 1 depicts the workflow of the experimental design used in this study.

### Characterization of MenSCs

MenSCs isolated from menstrual blood were characterized by multi-lineage differentiation and immunophenotyping. A considerable fraction of MenSCs were able to differentiate toward osteocyte, chondrocyte and adipocyte, as judged by specific stainings (Additional file 1: Fig. S1A). Cells grown in the absence of induction media failed to differentiate. MenSCs expressed markers of mesenchymal origin, but failed to express hematopoietic-specific markers. Unlike BMSCs, MenSCs expressed Oct-4 but were negative for SSEA-4 (Additional file 1: Fig. S1B). The results obtained here were almost the same with those we reported earlier [44, 47].

### Pro-inflammatory cytokines are needed to induce inhibitory capacity of MenSCs on T cell proliferation

**Fig. 2:**
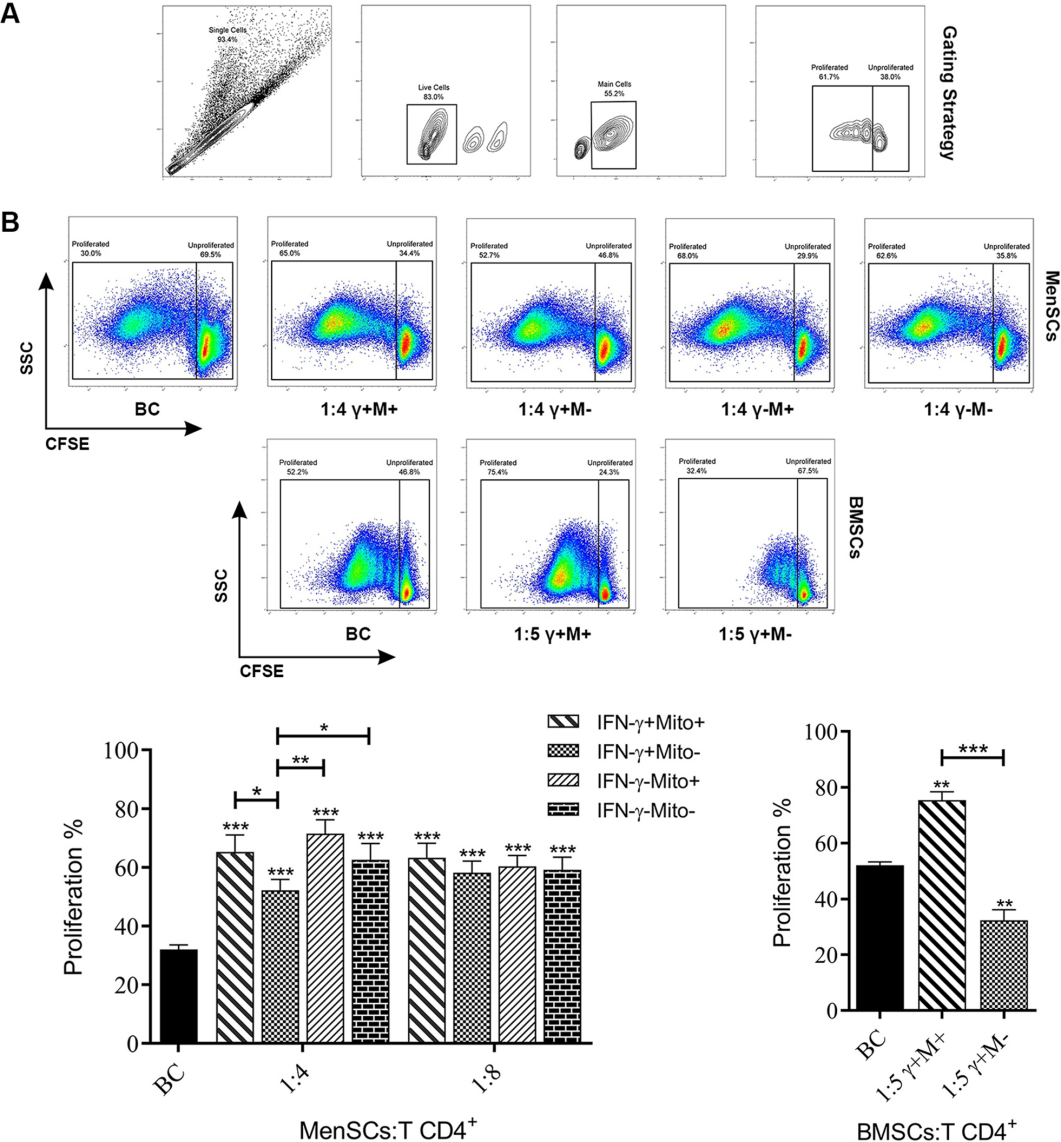
MenSCs induced T cell proliferation. MenSCs were either inactivated with Mitomycin C or remained intact. Some wells were also primed with IFN-γ prior to co-culture with T cells. CFSC-labeled purified CD4 + T cells were then co-cultured with MenSCs at different ratios and activated with T cell activation beads. The extent of T cell proliferation was then tested after five days by flow cytometry. The effect of BMSCs on T cell proliferation was also tested in the same conditions. Gating strategy is shown in panel (**A**). Representative flow cytometry dot plots and bar charts for MenSCs and BMSCs are shown in (**B**). T cells cultured in the absence of MenSCs or BMSCs served as biological control (BC). Data were expressed as mean ± SEM of 8 experiments. The signs (*), (**), (***), and (****) stand for *p* < 0.0332, *p* < 0.0021, *p* < 0.0002, and *p* < 0.0001, respectively

**Fig. 3:**
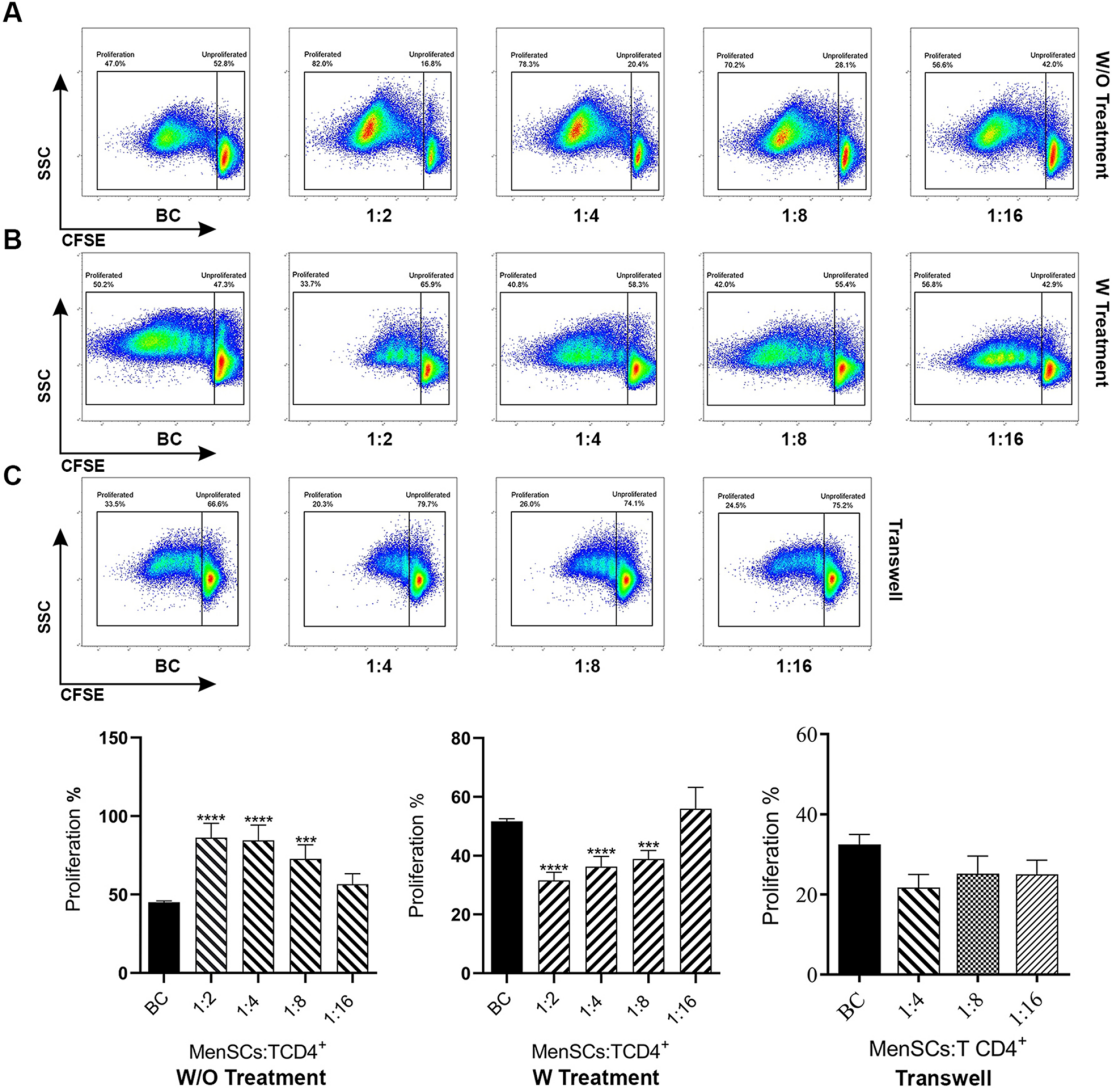
MenSCs pre-treated with IFN-γ and IL-1β inhibited T cell proliferation. MenSCs were either remained untreated (**A**) or primed with IFN-γ and IL-1β (**B**). CFSC-labeled purified CD4 + T cells were then co-cultured with MenSCs at different ratios and activated with T cell activation beads. Proliferation of T cells was then tested after five days by flow cytometry. In order to evaluate the effect of secretome of IFN-γ/IL-1β-treated MenSCs on CD4 + T cell proliferation, transwell plates were used with the aforesaid settings (**C**). T cells cultured in the absence of MenSCs served as biological control (BC). Data were expressed as mean ± SEM of 10 experiments. The signs (*), (**), (***), and (****) stand for *p* < 0.0332, *p* < 0.0021, *p* < 0.0002, and *p* < 0.0001, respectively

The capacity of MenSCs to modulate CD4 + T cell proliferation was tested at different conditions by flow cytometry. First, the impact of Mitomycin C and IFN-γ treatment of MenSCs on T cell proliferation was assessed. Untreated MenSCs were shown to increase the proliferation of CD4 + T cells compared to CD4 + T cells cultured alone (*p* < 0.0002). Mitomycin C inactivation of MenSCs had no effect on increased proliferation of CD4 + T cells co-cultured with MenSCs. IFN-γ treatment of MenSCs decreased their capacity to induce T cell proliferation compared to untreated MenSCs (*p* < 0.0332) (Fig. 2B). Expectedly, IFN-γ-treated BMSCs strongly inhibited T cell proliferation (*p* < 0.0021). However, the inactivation of BMSCs with Mitomycin C prior to IFN-γ-treatment reversed this effect and caused significant T cell proliferation (*p* < 0.0021). Based on these findings, all subsequent tests were done without Mitomycin treatment. Next, we tested the effect of MenSCs on CD4 + T cells proliferation at different ratios. As with our earlier report [48], we observed that MenSCs significantly promoted T cell proliferation compared to the biological control at 1:2–1:8 ratios (*p* < 0.0002–0.0001) (Fig. 3A). Regarding the simultaneous presence of multiple pro-inflammatory cytokines in early pregnancy decidua, the effect of simultaneous treatment of MenSCs with IFN-γ and IL-1β on T cell proliferation was tested at the next step. As depicted in Fig. 3B, simultaneous treatment of MenSCs with IFN-γ and IL-1β inhibited the proliferation of CD4 + T lymphocytes compared to the biological control (*p* < 0.0002–0.0001) at 1:2–1:8 ratios. In order to evaluate the effect of IFN-γ- and IL-1β-treated MenSCs on the proliferation of CD4 + T lymphocytes without cell–cell contact, transwell plates were used. As shown in Fig. 3C, although the proliferation of CD4 + T cells in the absence of cell–cell contacts was inhibited, the differences were not significant.

### Inhibition of PGE2 and IDO increased the proliferation of CD4 + T cells

**Fig. 4:**
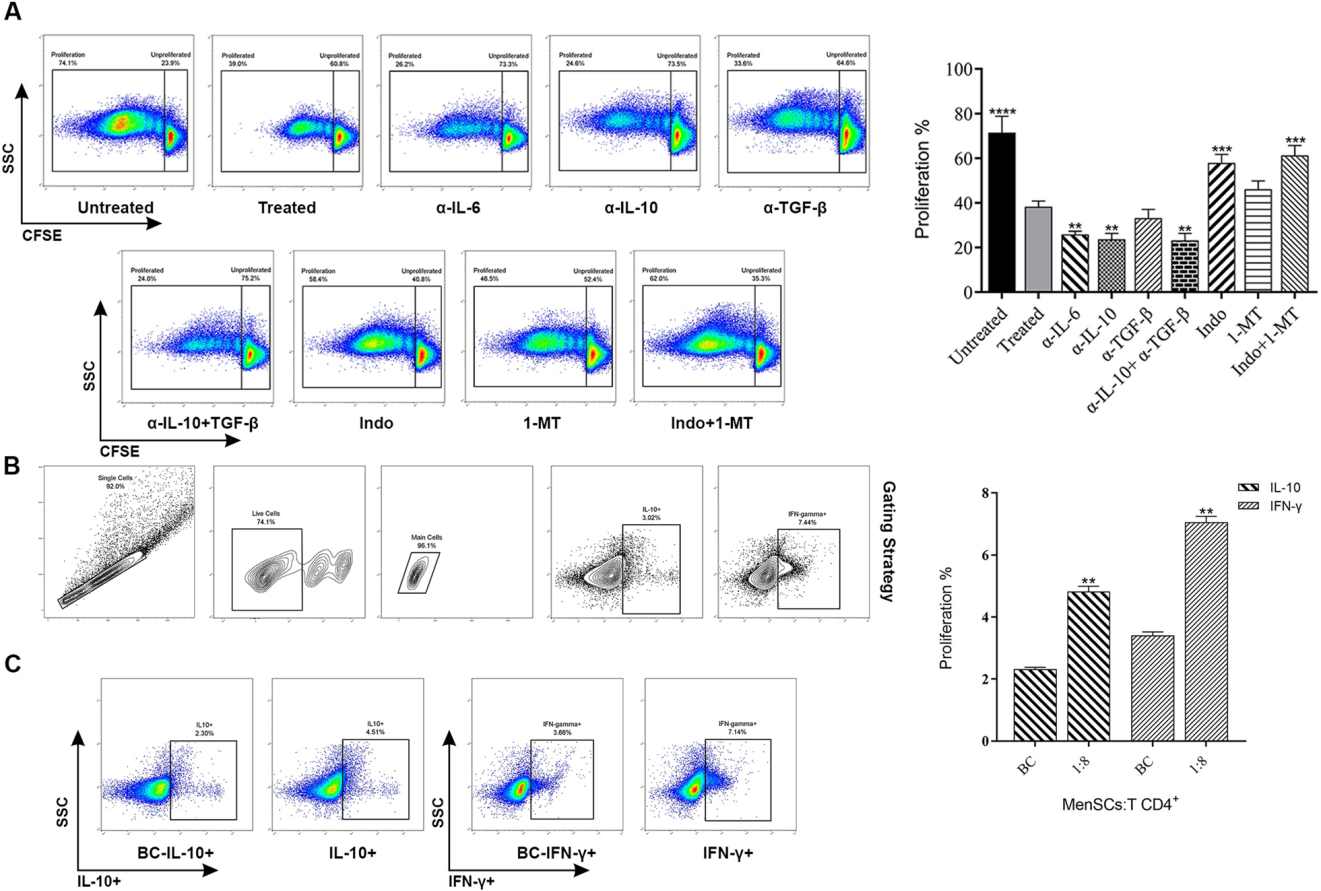
Inhibition of PGE2 and IDO increased the proliferation of CD4 + T cells. MenSCs were primed with IFN-γ and IL-1β before co-culture with CD4 + T cells. CFSC-labeled purified CD4 + T cells were then co-cultured with MenSCs at 1:8 ratio and activated with T cell activation beads. Different blockers including anti-IL-6, anti-IL-10, anti-TGF-β, indomethacin, 1 methyl tryptophan (1-MT) or their double combinations were added to the MenSCs-T cell co-cultures. The proliferation of T cells was then tested after five days by flow cytometry and compared to the extent of T cells which co-cultured withonly IFN-γ-/IL-1β-treated-MenSCs (**A**). Data were expressed as mean ± SEM of 12 experiments. The amount of IFN-γ and IL-10 in MenSCs-T cell co-culture was assessed by flow cytometry. Gating strategy is shown in panel (**B**) and representative flow cytometry dot plots are shown in (**C**). T cells cultured in the absence of MenSCs served as biological control (BC). The data obtained from each group were compared with biological control. The results were displayed as mean ± SEM of 8 different experiments. The signs (*), (**), (***), and (****) stand for *p* < 0.0332, *p* < 0.0021, *p* < 0.0002, and *p* < 0.0001, respectively

To explore the mediators responsible for inhibition of T cell proliferation by pre-treated MenSCs, different blockers including anti-IL-6, anti-IL-10, anti-TGF-β, indomethacin, 1 methyl tryptophan (1MT) or their double combinations were added to the MenSCs-T cell co-cultures at 1:8 ratio. Untreated MenSCs and MenSCs treated with IFN-γ and IL-1β served as untreated and treated controls, respectively. As illustrated in Fig. 4A, anti-IL-6, anti-IL-10, and combination of anti-IL-10 and anti-TGF-β failed to restore T cell proliferation. In contrary, treatment of MenSCs with indomethacin or double combination of indomethacin and 1-MT significantly restored T cell proliferation to the level observed in the treated control (p < 0.0002).

### IFN-γ/IL-1β-treated MenSCs induced IL-10 and IFN-γ production in CD4 + T cells

To see how MenSCs affect the balance of TH1/TH2, the levels of intracellular IL-10 and IFN-γ were measured in CD4 + T cells after co-culture with MenSCs at 1:8 ratio (MenSCs:CD4 + T cells). As shown in Fig. 4C, MenSCs significantly (*p* < 0.0021) increased the production of both cytokines in CD4 + T lymphocytes.

### MenSCs treated with IFN-γ- and IL-1β-induced Tregs

**Fig. 5:**
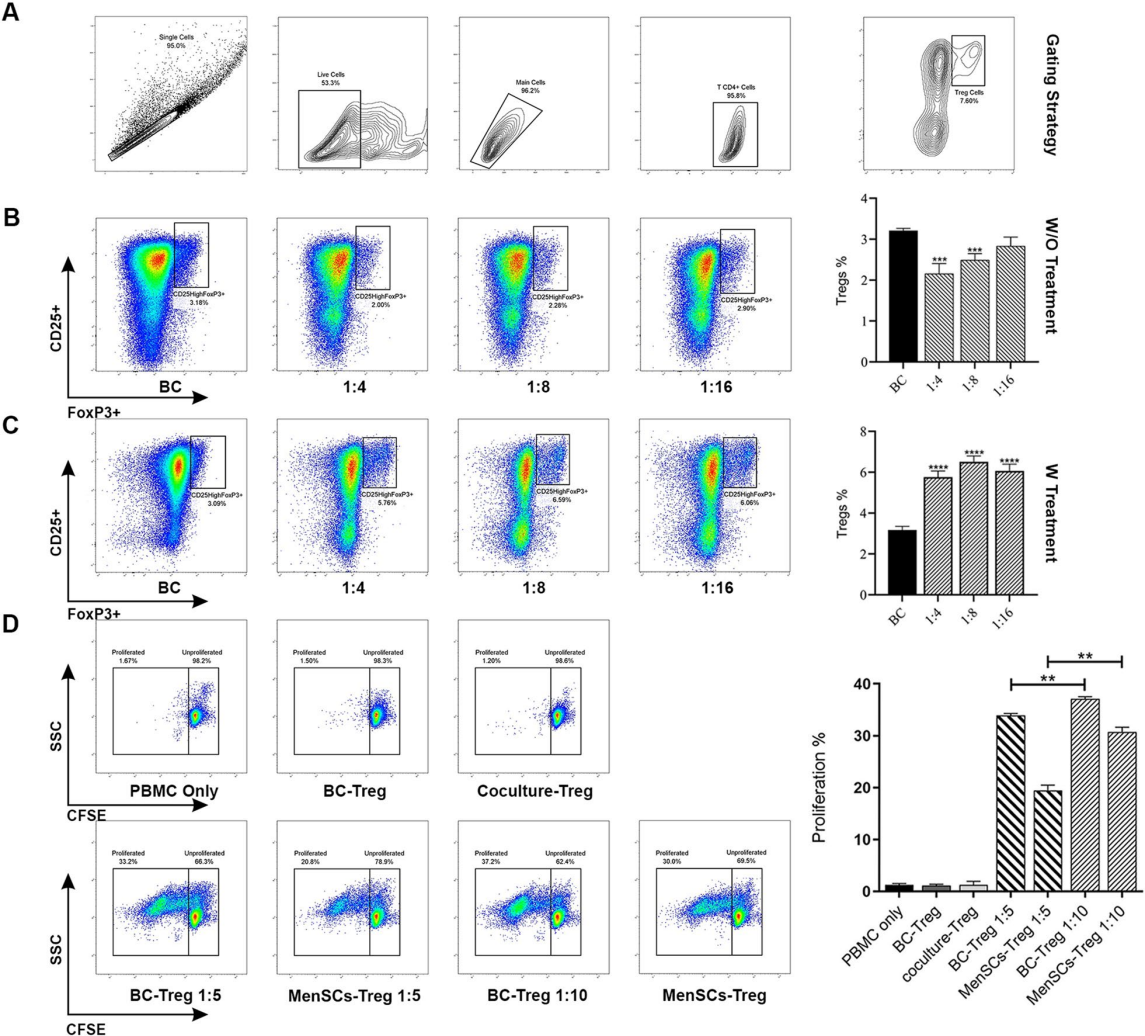
MenSCs pre-treated with IFN-γ- and IL-1β-augmented functional Treg generation. Purified CD4 + T cells were co-cultured with MenSCs at different ratios and activated with T cell activation beads. After five days, percent of CD4 + CD25^high^FOXP3 + Tregs was quantified by flow cytometry. Gating strategy for Tregs is shown in panel **(A)**. MenSCs were either remained untreated **(B)** or primed with IFN-γ and IL-1β **(C)**. T cells cultured in the absence of MenSCs served as biological control (BC). The data obtained from each group were compared with biological control, and the data were expressed as mean ± SEM of 12 experiments. IL-1β/IFN-γ-treated MenSCs were co-cultured with CD4 + T cells for five days and induced Tregs were then isolated by MACS. Tregs were added at 1:5 and 1:10 ratio to the CFSC-labeled autologous PBMCs cultures activated with activation beads and proliferation of PBMCs were measured after 4 days by flow cytometry **(D)**. Wells containing single PBMCs, Tregs (generated in the absence of MenSCs, BC-Treg), or MenSCs-induced Tregs (Coculture-Treg) served as negative controls. Co-cultures containing Tregs (generated in the absence of MenSCs) and PBMCs at ratio of 1:5 and 1:10 were considered as positive control. Results were displayed as Mean ± SEM of 6 different experiments. The signs (**) and (***) stand for *p* < 0.0021 and *p* < 0.0002, respectively

In the next step, the effect of untreated MenSCs on induction of Tregs was examined. T cells cultured alone served as biological control. As shown in Fig. 5B, untreated MenSCs decreased the frequency of Tregs at 1:4 and 1:8 co-culture ratios (*p* < 0.0002). The mean percentage of Tregs decreased from (3.21 ± 0.05) to (2.16 ± 0.24) and (2.49 ± 0.15) when T cells were co-cultured with untreated MenSCs at 1:4 and 1:8 ratios, respectively. Based on the results presented above, the effect of IFN-γ and IL-1β treatment of MenSCs on induction of Tregs was tested in the next step. As shown in Fig. 5C, MenSCs treated with IFN-γ and IL-1β significantly augmented Treg frequency at all co-culture ratios tested (*p* < 0.0001). The percentage of Tregs increased twofold when T cells were co-cultured with MenSCs pre-treated with pro-inflammatory cytokines.

### MenSCs-induced Tregs were functionally active to inhibit PBMC proliferation

Functional properties of Tregs generated in the presence of MenSCs were tested by inhibition of PBMCs proliferation. Wells containing single PBMCs, Tregs (generated in the absence of MenSCs), or MenSC-induced Tregs served as negative controls. Co-cultures containing Tregs (generated in the absence of MenSCs) and PBMCs were considered as positive control. Tregs were added at 1:5 and 1:10 ratio to the CFSC-labeled PBMC cultures activated with activation beads. All control wells showed only a negligible level of cell proliferation. As shown in Fig. 5D, MenSC-induced Tregs were able to significantly (*p* < 0.0021) inhibit the proliferation of PBMCs in both 1:5 and 1:10 ratios with a mean percentage of 19.50 ± 0.98 and 30.73 ± 0.89 compared to positive control groups 33.90 ± 0.40 and 37.1 ± 0.40, respectively.

### Blocking of IL-6, IL-10 and TGF-β decreased MenSCs ability to induce Tregs

**Fig. 6:**
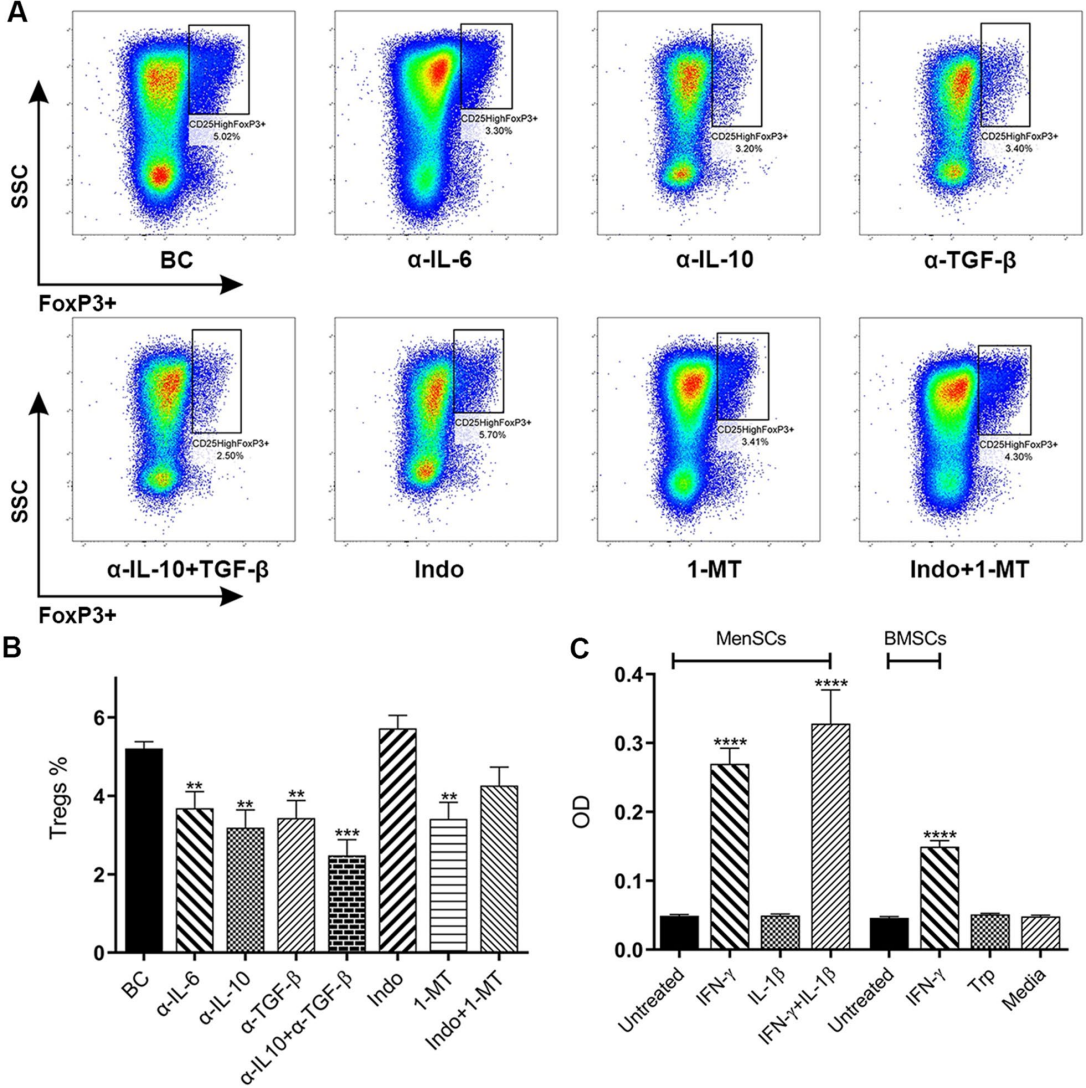
IL-6, IL-10, and TGF-β were involved in MenSCs-mediated Treg generation. MenSCs treated with IL-1β and IFN-γ were co-cultured with CD4 + T lymphocytes at 1:8 ratio and activated with T cell activation beads. Different blockers were added to the co-cultures. After five days, percent of CD4 + CD25^high^FOXP3 + Tregs was quantified by flow cytometry (**A**, **B**). T cells cultured in the absence of MenSCs served as biological control (BC). The data obtained from each group were compared with biological control, and the results were displayed as mean ± SEM of 8 experiments. Functional activity of IDO in MenSCs was tested using a colorimetric assay **(C)**. MenSCs were treated with IFN-γ and/or IL-1β for 48 h. IFN-γ-treated BMSCs served as positive control. Wells containing untreated cells or cells treated with Trp were considered as negative controls. Data were expressed as mean ± SEM of 16 different samples of MenSCs and BMSCs. The signs (*), (**), and (****) stand for *p* < 0.0332, *p* < 0.0021, and *p* < 0.0001, respectively. Indo: Indomethacin, 1-MT: 1-methyl tryptophan, Trp: Tryptophan

To find the cytokines involved in the induction of Tregs after co-culture with IFN-γ-/IL-1β-treated MenSCs, co-cultures at 1:8 ratios (MenSCs:CD4 + T cells) were treated with blocking antibodies against IL-6, IL-10, TGF-β, as well as indomethacin or 1-MT to inhibit PGE2 and IDO, respectively. Differentiation of CD4 + T cells toward Tregs in each group was compared to the control group containing the same co-culture without blocking agents. As illustrated in Fig. 6A and B, treatment with blocking antibodies against IL-6, IL-10, and TGF-β or 1-MT significantly decreased the percentage of Tregs (3.06 ± 0.35, 2.65 ± 0.38, 2.91 ± 0.40, and 3.40 ± 0.42, respectively, vs. 4.34 ± 0.14 in biological control group) (*p* < 0.0021). Co-treatment with anti-IL-10 and anti-TGF-β further decreased the percentage of Tregs (2.07 ± 0.33, *p* < 0.0002). However, inhibition of PGE2 did not affect Treg induction. Further, co-treatment with both indomethacin and 1-MT blockers had no significant effect on the induction of Tregs.

### Pre-treatment of MenSCs with inflammatory cytokines induced IDO activity

To study the activity of IDO secreted by MenSCs, these cells were pre-stimulated with IFN-γ as a potent inducer of IDO. We observed that the treatment of MenSCs with IFN-γ and IL-1β or IFN-γ alone could induce IDO activity (*p* < 0.0001) (Fig. 6C). Treatment of MenSCs with IL-1β alone did not induce IDO activity in these cells. As expected, IFN-γ-treated BMSCs produced a considerable amount of active IDO. Untreated cells or those treated with tryptophan did not exert a significant IDO activity.

## Discussion

Despite tremendous effort to unravel the immunology of reproduction in species with hemochorial placentation, the mystery of uterus as being a privileged site during pregnancy has not been resolved yet. Several hypotheses have been put forth so far and a dozen of mediators with immunosuppressive properties have been introduced to explain the state of uterine immune system hypo-responsiveness during pregnancy [4]. Nonetheless, with a closer view to the pathways and networks proposed in this regard, it became clear that many of them are interconnected and failure or dysfunction in one pathway might affect the proper functioning of the other pathways. Up to now, it remained unclear which component in the uterus is the main trigger of the delicate uterine immune regulatory network during pregnancy.

In recent years, the central role of mesenchymal stem cells in regulation of immune responses has been highlighted. BM-MSCs have been proved to exert an array of immune regulatory activities including inhibition of T cell proliferation and skewing T cell profile toward Tregs [26, 27]. MSCs from other sources such as those derived from adipose tissue also mediate generation of Tregs with immunosuppressive ability [28, 29]. Human endometrium is a dynamic tissue with ability for hundreds of cycles of growth, differentiation, and shedding during reproductive age. It contains a population of mesenchymal stem/stromal cell (eMSC) which shed during menstruation. While eMSC and MenSCs have several biological features with BM-MSCs, they also show some differences in immunophenotype, functional aspects, and differentiation capacities [49]. We recently showed that MenSCs are able to inhibit functional features of NK cells and TH17 differentiation to exert a pregnancy-friendly phenotype in this cell population [44, 47]. Here we were about to explore how biological features of Tregs, as the central cells in pregnancy tolerance, are affected by MenSCs.

We observed that MenSCs promoted the proliferation of CD4 + T cells in a dose-dependent manner. This results were in line with proliferation-promoting effect of MenSCs on NK and T cells [44, 48]. In our previous study, using protein microarray, we showed that MenSCs condition media contained considerably lower level of IGFBP1-4 (Insulin-like growth factor-binding protein) compared to BMSCs, leaving higher functional free form of IGFs to exert trophic activity [44]. We showed that blocking of IL-6 in the co-culture system resulted in reduced proliferation of T cells indicating supportive role of this cytokine for T cell proliferation. IL-6 has been shown to have a particular role in survival of resting [50] and activated T cells by preventing apoptosis in these cells through a mechanism, which is completely independent of IL-2 [51].

Several molecules have been reported to be involved in MSC-mediated immunoregulation. In this regard, pro-inflammatory cytokines exert a substantial role. IFN-γ alone or in combination with other pro-inflammatory cytokines such as TNF-α, IL-1α, or IL-1β have profound effects on stimulation of immunoregulatory phenotype in MSC [52, 53]. Therefore, in the next step we examined the potential effect of pro-inflammatory cytokines on MenSCs-induced T cell proliferation. We observed that IFN-γ treatment alone did not prevent the proliferation of T cells. However, combinational treatment of MenSCs with IFN-γ/IL-1β significantly reduced T cell proliferation. It was demonstrated that human embryonic stem cell-derived mesenchymal stromal cells (hES-MSCs) have no significant effect on lymphocyte proliferation despite pre-treatment with IFN-γ [54]. Depending on IFN-γ dose, umbilical cord blood mesenchymal stem cells have been reported to induce or suppress allogenic lymphocytes after pre-treatment with IFN-γ [55]. Responsiveness to pro-inflammatory cytokine treatment is largely dependent on MSC source. We showed that pre-treatment of BMSCs with the same concentration of IFN-γ resulted in substantially reduced T cell proliferation. Recent studies show that compared to BMSCs, MenSCs are less responsive to IFN-γ due to fewer expression of IFN-γR1 and IFN-γR2 [53]. Some studies show that adipose-derived stem cells (ASCs) could gain promoted inhibitory properties in the presence of IFN-γ, IL-1β, TNF-α, and IFN-α [56]. Our results showed that MenSCs have to be pre-treated with both IFN-γ and IL-1β to gain the capacity to suppress T cell proliferation. We also showed that, in contrast to conventional co-culture system, MenSCs pre-treated with IFN-γ/IL-1β did not significantly suppress T cell proliferation in a transwell system. Reportedly, IFN-γ along with TNF-α or IL-1β increased ICAM-1 and VCAM-1 expression and strengthened the attachment of MSCs and T cells in co-culture. Inhibition of these two molecules led to decreased suppressive effects of MSCs [57, 58]. In this regard, it seems that cell–cell contact plays a significant role in the inhibitory function of IFN-γ/IL-1β-treated MenSCs on T cell proliferation.

To investigate factors contributed in MenSC-induced suppression of T cell proliferation following IFN-γ/IL-1β treatment, IL-6, IL-10, TGF-β, PGE2 and IDO were blocked. We found that PGE2 was involved in suppression of T cell proliferation in co-culture system. PGE2 is a crucial player in chronic inflammation and could influence the functions of DC, NK and lymphocytes. High concentrations of PGE2 inhibit T lymphocyte proliferation through reducing both IL-2 and IL-2 receptor synthesis [59]. It is also shown that the inhibition of PGE2 reduced the immunosuppressive effects of human cord blood-derived mesenchymal stem cells (CB-MSCs) on T lymphocytes proliferation [60]. Simultaneous pre-treatment of MenSCs with IFN-γ and IL-1β resulted in increased expression of COX-2 [53]. Moreover, IFN-γ and IL-1β are well-known inducers of PGE2 because both of them can stimulate COX-2 mRNA expression [61, 62]. Inhibition of IDO had not a significant effect on T cell proliferation. This datum is in line with Gieske et al. that reported anti-proliferative properties of human multipotent mesenchymal stromal cells (MSCs) are independence of IDO [63]. Similarly, inhibition of TGF-β alone showed a negligible effect on the proliferation of co-cultured CD4 + T cells. This finding is in line with what reported earlier for umbilical cord MSCs [61]. However, we showed simultaneous inhibition of IL-10 and TGF-β or inhibition of IL-10 alone could significantly reduce the proliferation of CD4 + T cells. In this regard, Harizi and Gualde reported that IL-10 can inhibit COX-2 expression at mRNA and protein levels, which leads to inhibition of PGE2 production in BMDC (bone marrow-derived DC) [64]. As mentioned above, PGE2 plays a pivotal role in suppressive activity of MSCs, therefore by blocking IL-10, PGE2 production could be enhanced leading to further suppression of T cell proliferation. Nonetheless, based on the complex nature of in vivo microenvironment, it is evident that none of these molecules has an exclusive role and that MSC-mediated immunoregulation is mediated by a combination of several molecules, which do not exert their effects in the same direction.

One potential way that MSCs cells could inhibit proliferation of immune cells is their ability to induce Treg cells. Therefore, we next aimed to investigate the effect of MenSCs on induction of Treg lymphocyte population. We found that in the absence of inflammatory cytokines, MenSCs did not induce Tregs, neither did they decrease their frequency. MenSCs can secrete a baseline levels of IL-6 and TGFβ in proinflammatory environment [65], which could be increased in an allogeneic cell culture system. In a mouse GVHD model, the inhibition of the IL-6 signaling pathway markedly reduced pathologic damage through a significant increase in the absolute number of Tregs [66]. Indeed, IL-6 could induce STAT3-mediated T cell production of IL-10, a cytokine with Treg induction capacity [67]. Similarly, our results showed that higher levels of IL-10 were produced in MenSCs-T cell co-culture supernatant and that inhibition of IL-10 or TGF-β significantly inhibited the induction of Tregs. The crucial impact of these cytokines on Treg generation has already been reported. Moreover, induction and propagation of Tregs by MSCs are closely related to IL-10 and TGF-β cytokines [68]. We showed that treatment of MenSCs with IFN-γ and IL-1β led to increased level of IDO generation, a finding which is in line with our previous findings [46]. IDO dominantly controls the generation and functional status of Tregs in response to inflammatory stimuli, which could potentially lead to generation of Tregs.

Finally, we showed that Tregs generated in the presence of MenSCs are functionally active and could prevent proliferation of PBMCs activated with anti-CD3/CD28 antibodies and IL-2. Similar results have also been reported for other sources of MSCs either in mixed lymphocyte reaction [27, 69–71]. It seems that higher immunosuppressive phenotype in Tregs generated in the presence of MSCs is due to the induction of these cells in an inflammatory milieu.

## Conclusion

Collectively, we showed here that, in contrast to other sources of stem cells, MenSCs have a supportive effect on the proliferation of CD4 + T lymphocytes and reduced percentage of Tregs unless they were first primed with pro-inflammatory cytokines. Pro-inflammatory cytokines-primed MenSCs could enhance functionally active Tregs and suppress T cell proliferation, a finding which support their role as the main regulator of the endometrial immune microenvironment during pregnancy.

## Supplementary Information

**Additional file 1: Fig. S1.** Differentiation of MenSCs into adipocytes, osteoblasts and chondrocytes. MenSCs were differentiated toward adipocytes, osteoblasts and chondrocytes and the extend of differentiation was evaluated using Alizarin red, Alcian blue and Oil Red O staining, respectively. Undifferentiated cells served as controls (**A**). Immunophenotyping of MenSCs was carried out using a panel of mesenchymal and hematopoietic markers (**B**).

## Data Availability

All the datasets used or analyzed in this study are available from corresponding author on reasonable request.

## Abbreviations

1-MT: 1-methyl tryptophan
ASCs: adipose-derived stem cells
AT-MSCs: adipose tissue-derived multipotent stromal cells
BMDC: bone marrow-derived DC
BMSCs: bone marrow mesenchymal stem cells
CB-MSCs: cord blood-derived mesenchymal stem cells
CFSE: 5,6-carboxyfluorescein diacetate succinimidyl ester
COX-2: cyclooxygenase-2
DC: dendritic cell
DSCs: decidua-derived stem cells
eMSCs: endometrial stromal/stem cells
GVHD: graft versus host disease
HBV: hepatitis B virus
HCV: hepatitis C virus
hES-MSCs: human embryonic stem cell-derived mesenchymal stromal cells
HIV: human immunodeficiency virus
ICAM-1: intercellular adhesion molecule 1
IDO: indoleamine-pyrrole 2,3-dioxygenase
IFN-γ: interferon gamma
IGFs: insulin-like growth factors
IGFBP: insulin-like growth factor-binding protein
IL-1β: interleukin 1 beta
IL-2: interleukin 2
IL-6: interleukin 6
IL-10: interleukin 10
MenSCs: menstrual stromal/stem cells
MLR: mixed lymphocyte reaction
NK: natural killer cell
Oct-4: octamer-binding transcription factor 4
PBMCs: peripheral blood mononuclear cells
PGE2: prostaglandin E2
SSEA-4: stage-specific embryonic antigen 4
STAT3: signal transducer and activator of transcription 3
TGF-β: transforming growth factor beta
TH1: type 1 T helper cells
TH2: type 2 T helper cells
TNF-α: tumor necrosis factor alpha
Tregs: regulatory T cells
VCAM-1: vascular cell adhesion protein 1
