# Supplementary figures and images for: Endometrial mesenchymal stem/stromal cells: The Enigma to code messages for generation of functionally active regulatory T cells
Source: Stem Cell Res Ther. 2021 Oct 9;12:536. doi: 10.1186/s13287-021-02603-3 (PMC8502414; doi:10.1186/s13287-021-02603-3)

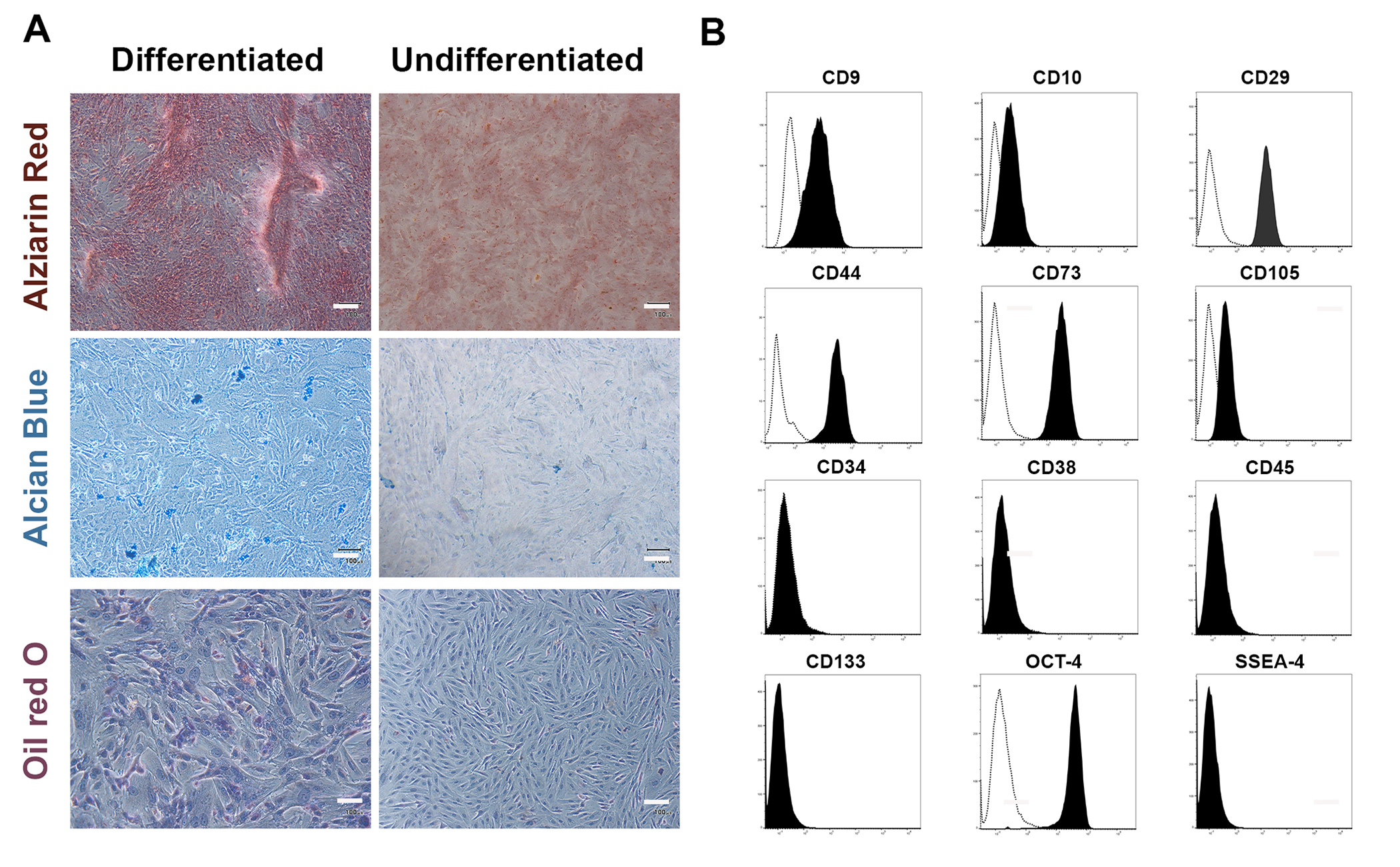

Supplement: Supplementary file 1 — Additional file 1: Fig. S1. Differentiation of MenSCs into adipocytes, osteoblasts and chondrocytes. MenSCs were differentiated toward adipocytes, osteoblasts and chondrocytes and the extend of differentiation was evaluated using Alizarin red, Alcian blue and Oil Red O staining, respectively. Undifferentiated cells served as controls (A). Immunophenotyping of MenSCs was carried out using a panel of mesenchymal and hematopoietic markers (B). [file 13287_2021_2603_MOESM1_ESM.jpg]
